# SARS-CoV-2 wildlife surveillance surrounding mink farms in British Columbia, Canada

**DOI:** 10.14745/ccdr.v48i06a03

**Published:** 2022-06-09

**Authors:** Talia Strang, Logan Flockhart, Caeley Thacker, Helen Schwantje, Catherine Soos, Antonia Dibernardo, L Robbin Lindsay, Nikki Toledo, Kaela Beauclerc, Erin Fraser, Natalie Prystajecky, Chelsea Himsworth

**Affiliations:** 1 BC Centre for Disease Control, Vancouver, BC; 2 Surveillance Division, Public Health Agency of Canada, Saskatoon, SK; 3 Ministry of Forests, Lands, Natural Resource Operations and Rural Development, Victoria, BC; 4 Wildlife Health, Environment and Climate Change Canada, Ottawa, ON; 5 National Microbiology Laboratory, Public Health Agency of Canada, Winnipeg, MB; 6 Ministry of Northern Development, Mines, Natural Resources and Forestry, Peterborough, ON; 7 School of Population and Public Health, University of British Columbia, Vancouver, BC; 8 Department of Pathology and Laboratory Medicine, University of British Columbia, Vancouver, BC; 9 Ministry of Agriculture, Food, and Fisheries, Abbotsford, BC; 10 BC Regional Centre, Canadian Wildlife Health Cooperative, Abbotsford, BC

**Keywords:** SARS-CoV-2, American mink, wildlife surveillance, physical trapping, camera trapping

## Abstract

**Background:**

Severe acute respiratory syndrome coronavirus 2 (SARS-CoV-2) can infect many wild and domestic animal species. Farmed American mink (*Neovison vison*) are particularly susceptible to infection. Outbreaks of SARS-CoV-2 were detected in farmed mink on three mink farms in British Columbia (BC), Canada between December 2020 and May 2021. In BC, mink farm density and proximity to wildlife habitats increase transmission risks from infected farmed mink. The objective of this study is to investigate the risk of SARS-CoV-2 spreading to and from wildlife in the area surrounding infected mink farms in BC, Canada, as well as to compare the effectiveness of physical and camera trapping surveillance methodologies.

**Methods:**

A combination of physical and camera trapping was used on and around three BC mink farms with active SARS-CoV-2 infections between January 22, 2021, and July 10, 2021. Samples from trapped animals, including escaped farmed mink, were tested for SARS-CoV-2. Camera images from one mink farm were reviewed to determine species and proximity to the mink barn.

**Results:**

Seventy-one animals of nine species were captured and sampled. Three captured mink tested positive for SARS-CoV-2 by polymerase chain reaction and serology; the remaining samples were negative for SARS-CoV-2. Genotyping of the three positive mink indicated these were domestic (vs. wild) mink. A total of 440 animals of 16 species were photographed at the one farm where cameras were deployed.

**Conclusion:**

Detection of SARS-CoV-2 in escaped farmed mink is concerning and demonstrates the potential for transmission from farmed mink to wildlife, particularly given the observation of wildlife known to be susceptible to SARS-CoV-2 near infected mink farms. Combined use of physical and camera trapping contributed to the breadth of the results and is strongly recommended for future surveillance.

## Introduction

The ongoing coronavirus disease 2019 (COVID-19) pandemic caused by severe acute respiratory syndrome coronavirus 2 (SARS-CoV-2) is responsible for substantial morbidity and mortality of humans globally ((1)). SARS-CoV-2 is zoonotic in origin, but once the spillover to humans occurred the course of the pandemic has been driven almost entirely by human-to-human transmission. Natural infections of SARS-CoV-2 have been detected in a wide range of animals, including castorids (Sino-Mongolian beaver) ((2)), cervids (white-tailed deer) ((3)), cricetids (hamsters) ((4)), felids: (domestic cats) ((5,6)), cougars, fishing cats, lions, Canada lynx (7)), snow leopards, tigers ((8–10)), domestic dogs and cats ((5,6)), gorillas, hippopotamus ((11)), mustelids (American mink) ((12–16)), Asian small-clawed otters and ferrets ((17)), procyonids (coatimundi), spotted hyenas ((18)) and viverrids (bearcats) ((7)) (**Table 1**).

**Table 1:** Susceptibility of observed species to severe acute respiratory syndrome coronavirus 2^a^

Order	Family	Species	Susceptibility to SARS-CoV-2
Carnivora	Canids	Coyote (*Canis latrans*)	Unknown
	Felids	Cat (*Felis catus*)	High
	Mustelids	Mink (*Neovison vison*)	High
		Otter (*Lontra canadensis*)	High
	Procyonids	Raccoon (*Procyon lotor*)	Low
Lagomorpha	Leporids	Rabbit (*Sylvilagus* sp.)	Yes
Rodentia	Castorids	Beaver (*Castor canadensis*)	Yes
	Murids	Rat (*Rattus* sp.)	Unknown
Anseriformes	Anatids	Mallard duck (*Anas platyrhynchos*)	Unknown
		Wood duck (*Aix sponsa*)	Unknown
Galliformes	Phasianids	Chicken (*Gallus gallus domesticus*)	None
Passeriformes	Corvids	Crow (*Corvus brachyrhynchos*)	Unknown
	Sturnids	Starling (*Sturnus vulgaris*)	Unknown
Pelecaniformes	Ardeids	Blue heron (*Ardea herodias*)	Unknown
Strigiformes	Strigids	Barred owl (*Strix varia*)	Unknown

^a^ Based on information from Animals and COVID-19

Transmission of SARS-CoV-2 in American mink (*Neovison vison*) is of particular concern. Mink are highly susceptible to the virus, and the virus has been found to undergo mutation at a higher rate in mink than in humans ((19)). Mink are farmed globally in high density environments, and there is evidence of transmission of SARS-CoV-2 from mink to humans and vice versa (20–23)). These factors increase the transmission risk of SARS-CoV-2 in mink, potentially leading to viral mutations and the emergence of variants of concern for human health.

The SARS-CoV-2 in mink also poses a risk to wildlife. Indeed, free-ranging infected mink have been detected in the United States (US) and Spain and, in both countries, these animals were believed to have escaped from nearby infected farms ((24)). Mink have also been shown to transmit SARS-CoV-2 to domestic dogs and cats in and around the farm environment ((12,24)) and other diseases, such Aleutian disease, which have been shown to spillover from infected mink farms into wildlife populations ((25)). For this reason, the World Organisation for Animal Health ((26)) and US Department of Agriculture ((27)) have recommended surveillance for SARS-CoV-2 in wildlife potentially exposed to domestic animal reservoirs of the virus and Environment and Climate Change Canada have issued national guidelines recommending surveillance of wildlife around infected mink farms ((24)). This surveillance is focused on trapping and testing of target wildlife species in a 1–3 km range around infected farms and aligns with similar surveillance programs in the US ((28)).

In the province of British Columbia (BC), the mink farming industry is regulated by the BC Ministry of Agriculture, Food and Fisheries. In December 2020, there were nine active farms licensed in BC, all located in the Lower Mainland region. The SARS-CoV-2 was detected in farmed American mink on two mink farms in BC in December 2020 (Farm 1 and Farm 2) and on one farm in May 2021 (Farm 3). The original source of SARS-CoV-2 in mink on two of three affected mink farms was COVID-19 infections in mink farm workers. The source of infection of the third mink farm was not determined conclusively; however, genetic sequencing indicated that the strain was similar to human cases of COVID-19 in the local community at the time of detection (*N. Prystajecky, personal communication, 2021*).

The detection of SARS-CoV-2 on the mink farms raised concerns about spread to wildlife in the surrounding area. It is of note that the aforementioned Environment and Climate Change Canada surveillance guidelines were published in November 2021, after surveillance around the infected farms was completed; however, the methods employed (including live trapping and SARS-CoV-2 testing of wildlife around farms, genetic testing of free-ranging mink and supplementing trapping data with information gleaned from camera footage) are largely aligned with the national recommendations.

Here we report on the results of wildlife surveillance for SARS-CoV-2 around the three infected mink farms in BC with a view to assessing the risk of the virus spreading to and from wildlife in the vicinity of mink farms. Furthermore, the broader purpose of this analysis is to compare physical and camera trapping surveillance strategies, and ultimately to inform future wildlife surveillance strategies to optimise risk assessments for both public health and wildlife health.

## Methods

### Physical trapping

The outbreak on Farm 1 lasted from December 2, 2020, to February 24, 2021, and the outbreak on Farm 2 lasted from December 23, 2020, to December 26, 2020, when the producer opted to euthanize the whole herd. Ring surveillance was used around Farm 1 and Farm 2. Seventy traps were placed in a three-kilometre perimeter surrounding the two farms from January 22, 2021, to March 19, 2021. Target species were selected based on what species were known to be present in the area and what was known about species susceptibility at the time. Primary target species included feral cats (*Felis catus*), escaped domestic mink (*N. vison*) and wild mustelids such as wild mink and otters (*Lontra canadensis*). Raccoon (*Procyon lotor*), striped skunk (*Mephitis mephitis*), Virginia opossum (*Didelphis virginiana*) and bobcat (*Lynx rufus*) were also expected in the areas and considered target species but likely presented lower likelihood of SARS-CoV-2 carriage. White-tailed deer (*Odocoileus virginianus*) were not targeted as the extent of their susceptibility was not known at the time of sampling. A mixture of live and kill traps (Tomahawk Durapoly small, 120 Conibear, 330 Conibear, Havahart 1079, Havahart 1081) were used based on trapper experience and target species. Where live traps were used, the animal was then humanely euthanized. Note that the target species were used to inform the trapping methodology; however, all animals trapped, regardless of species, were included in the surveillance sample, including opportunistically collected roadkill animals. Live and kill traps were selected to meet certification and requirements of the *Agreement on International Humane Trap Standards*. All physical trapping was carried out by experienced wildlife trappers who were familiar with the geographical area and the patterns of local wildlife.

The outbreak on Farm 3 lasted from April 2, 2021, to February 11, 2022. Risk-based surveillance was implemented on Farm 3 by focusing on mustelids (the species group in the area considered most susceptible to SARS-CoV-2) within and immediately adjacent to the farm. This approach was adopted because trapping occurred during the breeding season; therefore, it was critical to target specific higher-risk species and exclude pregnant and lactating female. Twenty-four live traps were placed from June 23, 2021, to July 10, 2021, in three areas: on farm property (n=6); around the perimeter of the farm property (n=6); and in adjacent suitable mustelid habitat (n=12) that consisted of farmland and river habitat. Animals were assessed in the live traps and those that were neither pregnant nor lactating were humanely euthanized.

Samples collected from euthanized animals included nasal swabs for SARS-CoV-2 polymerase chain reaction (PCR), which were placed in viral transport medium prior to testing at the Animal Health Centre, Abbotsford. Whole blood for serological analysis was collected by saturating the length of Nobuto filter strips (Fisher Scientific, Waltham, Massachusetts, US) with cardiac blood. These were air dried and stored in individual envelopes at 4°C until shipped to the National Microbiology Laboratory, Winnipeg, Manitoba for testing. Skin samples were collected from three SARS-CoV-2-positive mink for microsatellite genotyping to investigate their ancestry (i.e. domestic vs. wild) and analyzed at the Wildlife Genetics Lab of the Wildlife Research and Monitoring Section, Ontario Ministry of Northern Development, Mines, Natural Resources and Forestry, Peterborough, Ontario.

### Severe acute respiratory syndrome coronavirus 2 polymerase chain reaction testing

Approximately 1.5 ml nasal swab in Virus Transport Media (VTM) was clarified by centrifugation at 2,000 g for two minutes. Viral ribonucleic acid (RNA) was isolated using the Applied Biosystems Incorporated MagMax-96 Express magnetic particle processor (ThermoFisher Scientific, Waltham, Massachusetts, US) with the MagMax™-96 Viral RNA Isolation Kit (ThermoFisher, catalog number: AM1836) as per kit instructions. The MagMax program (AM1836_DW_v50) was available on the ThermoFisher website (thermofisher.com). Primers and probe that target the E gene to create a 113-base pair (bp) amplicon were used to detect SARS-CoV-2. Forward primer 5’-ACAGGTACGTTAATAGTTAATAGCGT-3’; probe 5’- FAM-ACACTAGCCATCCTTACTGCGCTTCG-BHQ1- 3’, reverse primer 5’-ATATTGCAGCAGTACGCACACA -3’. Reaction concentrations of the SARS-CoV-2 primers and probe were 800 nM and 200 nM, respectively. An enterovirus exogenous PCR control (Asuragen, catalog number: 42050) was spiked in the RNA isolation step and the 61 bp amplicon was detected with the following primers and probe: forward primer 5’- ATGCGGCTAATCCCAACCT -3’; probe 5’- VIC-CAGGTGGTCACAAAC -MGBNFQ -3’; and reverse primer 5’- CGTTACGACAGGCCAATCACT -3’ (VIC and MGBNFQ are proprietary dyes to Applied Biosystems). The reaction concentration for the enterovirus primers and probe were 200 nM each. The AgPath-ID™ One-Step RT-PCR Reagents was used as per kit instructions (ThermoFisher, catalog number: 4387391): 5 µl of extracted RNA template was added to the master mix. Real-time PCR (RT-PCR) was performed on the Applied Biosystems 7500 Fast Real-Time PCR System thermocycler using with the following amplification profile: one cycle of 50°C, 30 minutes; one cycle of 95°C, one minute; 40 cycles of 95°C, 15 seconds and 60°C, one minute. Change in fluorescence was recorded at the elongation step of each cycle.

### Severe acute respiratory syndrome coronavirus 2 serology

Serological testing of whole blood was conducted using the GenScript cPass™ SARS-CoV-2 Neutralization Antibody Detection Kit (catalog number: L00847, GenScript US, Inc. Piscataway, New Jersey, US) according to manufacturer's protocol. Samples with more than 30% inhibition were considered positive for SARS-CoV-2 neutralizing antibodies. To minimize possible risk of exposure to the zoonotic pathogen, *Francisella tularensis,* by laboratory staff, serum samples were not collected from beavers as per laboratory guidelines at the BC Animal Health Centre.

### Genotyping of mink

Microsatellite profiling of free-ranging mink samples followed the procedure detailed in Beauclerc *et al.* ((29,30)) with minor modifications. Briefly, whole genomic DNA was extracted from approximately 10 mg of muscle with the E.Z.N.A.^®^ Tissue DNA Kit (Omega Bio-Tek) and quantified with PicoGreen dye (Invitrogen). Samples were amplified at 15 microsatellite loci in 2 multiplexes, each consisting of 12 μL reactions with 1 ng DNA, primer labels and concentrations as shown in **Table A1****.** Genotyping was performed on an ABI 3730 with GeneScan 500HD ROX (Applied Biosystems). Fragments were scored automatically in GeneMarker v.2.6.4 (SoftGenetics) and verified by eye; ambiguous alleles were reamplified.

### Camera trapping

In addition to physical trapping, more in-depth camera trapping was implemented on Farm 3 from February 7, 2021, to July 25, 2021, based on experience from Farm 1 and Farm 2. Camera trapping was utilized to gather more information on the presence of animals and their use of the habitats surrounding mink farms and to avoid physical disruption during the breeding season of relevant species. This involved the placement of 11 wildlife cameras inside the fenced area surrounding the mink barn (n=1), outside but adjacent to the fenced barn (n=4) and near the river adjacent to the perimeter of the farm property (n=6). Images of animals captured on camera were then analyzed visually. The species present in each image was identified based on morphology.

## Results

### Physical trapping

A total of 71 animals of nine different species were trapped, including 63 from Farm 1 and Farm 2 and 8 from Farm 3 (**Table 2**). All trapped animals appeared healthy upon visual examination. Two trapped cats were observed as acting aggressively. Several of the trapped species are known to be susceptible to infection with SARS-CoV-2, specifically domestic cats, mink, otters, rabbits and raccoons ((31)). The susceptibility for many other species is currently unknown (Table 2) ((31)).

**Table 2:** Species captured during physical trapping around severe acute respiratory syndrome coronavirus 2-infected mink farms in British Columbia (n=71)

Order	Family	Species	Number of captures	Percent of total
Carnivora	Felids	Cat (*Felis catus*)	5	7
	Mustelids	Mink (*Neovison vison*)	12	17
		Otter (*Lontra canadensis*)	1	1
	Procyonids	Raccoon (*Procyon lotor*)	4	6
Didelphimorphia	Didelphids	Opossum (*Didelphis virginiana*)	6	8
Rodentia	Castorids	Beaver (*Castor canadensis*)	9	13
	Cricetids	Muskrat (*Ondatra zibethicus*)	6	8
	Murids	Rat (*Rattus* sp.)	10	14
	Sciurids	Grey squirrel (*Sciurus carolinensis*)	18	25

Mink were assigned to their population of origin using Bayesian assignment tests in STRUCTURE v.2.2 for assumed number of clusters (K) of two, as described in Bowman *et al.* (30,32)). Previously analysed samples, consisting of domestic and free-ranging samples from Ontario, Nova Scotia and Prince Edward Island (n=902), provided the reference dataset within which the new samples were analysed (29,30)). Membership in a cluster used the average ancestry coefficient (q): individuals with q>0.8 were assigned to a single cluster, while those with q<0.8 were considered hybrids ((33)).

### Severe acute respiratory syndrome coronavirus 2 polymerase chain reaction, serology and genotyping of mink

All sampled animals were negative for SARS-CoV-2 using PCR and serology, with the exception of three mink trapped on the property of Farm 3 outside the barrier fence that were both PCR-positive and had antibodies against SARS-CoV-2. These three mink were genotyped and genotyping highly assigned these mink to the domestic cluster (q=0.94–0.99), indicating that they were domestic (vs. wild) mink that had likely escaped from their cages. Note that none of the other trapped mink was genotyped.

### Camera trapping

There were 440 camera images showing 1 or more animals of 1 of 16 species (**Table 3**). Of note, cats and crows were observed inside the barrier fence with access to the mink barn. Additionally, some species were observed near the mink barn but outside the barrier fence, specifically coyotes, cats, mink, rabbits, crows, starlings and owls (Table 3).

**Table 3:** Species observed during camera trapping around Farm 3, (n=440)

Order	Family	Species	Number of observations	Percent of observations	Percent within proximity of mink barn	
					%	n
Carnivora	Canids	Coyote (*Canis latrans*)	144	33	61	n=88/144
	Felids	Cat (*Felis catus*)	59	13	49	n=29/59
	Mustelids	Mink (*Neovison vison*)	5	1	40	n=2/5
		Otter (*Lontra canadensis*)	3	<1	0	n=0/3
	Procyonids	Raccoon (*Procyon lotor*)	7	2	0	n=0/7
Lagomorpha	Leporids	Rabbit (*Sylvilagus* sp.)	14	3	100	n=14/14
Rodentia	Castorids	Beaver (*Castor canadensis*)	11	3	0	n=0/11
	Murids	Rat (*Rattus* sp.)	2	<1	0	n=0/2
Anseriformes	Anatids	Mallard duck (*Anas platyrhynchos*)	21	5	0	n=0/21
		Wood duck (*Aix sponsa*)	26	6	0	n=026
Galliformes	Phasianids	Chicken (*Gallus gallus domesticus*)	1	<1	0	n=0/1
Passeriformes	Corvids	Crow (*Corvus brachyrhynchos*)	110	25	22	n=24/110
	Sturnids	Starling (*Sturnus vulgaris*)	7	2	43	n=3/7
Pelecaniformes	Ardeids	Blue heron (*Ardea herodias*)	21	5	0	n=0/21
Strigiformes	Strigids	Barred owl (*Strix varia*)	2	<1	100	n=2/2
Bird—unknown classification			7	2%	14	n=1/7

Note: A severe acute respiratory syndrome coronavirus 2-infected mink farm in British Columbia

It is particularly of interest that three mink were observed outside of the barrier fence surrounding the mink barn. While it is not certain that these were escaped farmed mink, it is very likely, given that mink trapped in similar locations were genotyped as domestic mink.

## Discussion

Wildlife surveillance involving physical and camera trapping surrounding mink farms in BC infected with SARS-CoV-2 identified 71 animals of nine different species from physical trapping and 440 observations of 16 different species from camera trapping. Three mink trapped on one farm property were PCR-positive and seropositive for SARS-CoV-2. Additionally, mink were observed on camera that were likely escaped farmed mink.

The observation of wildlife in proximity to infected mink farms, particularly those species known to be susceptible to SARS-CoV-2, demonstrates the risk of transmission from farmed mink to wildlife. Of particular concern was the capture of three escaped farmed mink that tested positive for SARS-CoV-2, as well as the observation of mink on camera footage (although it could not be confirmed whether these animals represent additional escapees). These were consistent with findings from the US and Spain in which SARS-CoV-2 surveillance was conducted around infected mink farms ((28,34)). In those studies, exposure and infection was only detected in free-ranging mink that were thought to have escaped from infected farms ((28,34)). For the infected mink caught on the farm property in this study, it is problematic that they were able to escape from the caging and barrier fence; however, being found within the farm property is less of a concern than if they had been found outside the farm as they are less likely to have had extensive contact with wildlife.

Feral cats and crows were observed (via cameras) inside the fence in the immediate area of the mink barn. Continued surveillance of these species is prudent, particularly for cats as they are known to be susceptible to SARS-CoV-2, appear to have greater access to mink barns compared with other species and can often be in close contact with humans. Furthermore, a previous study reported that a feral cat on a mink farm in the Netherlands tested positive for SARS-CoV-2 (12)). In combination, these factors could allow cats to facilitate interspecies transmission of SARS-CoV-2 ((35)). Continued surveillance of birds should also be considered. While birds are not known to carry or transmit the virus to conspecifics, other wildlife or humans, they may act as fomites through contact with and carriage of contaminated material or surfaces (36)). Additionally, surveillance of wild ungulates should be considered due to their high susceptibility to SARS-CoV-2 infection and transmission (31)). Although outside the barrier fence, other wildlife known to be susceptible to SARS-CoV-2 (e.g. raccoon, rabbit, otter and beaver) ((31)) were trapped or observed in close proximity to the mink farms. Overall, although no spillover from farmed mink to any wildlife species was detected, the potential for farmed mink to come into close contact with wildlife species or feral and domestic animals and transmit SARS-CoV-2 to wildlife, via aerosol transmission, exists.

This implementation of different surveillance methods demonstrated that both physical and camera trapping provided important information, and the conclusions drawn were strengthened by the combined data. Physical trapping using ring surveillance was beneficial when little was known about SARS-CoV-2 and the potential for spillover. This is because a greater number of animals from more diverse species were caught. Once more information was known, a more focused approach at one facility using risk-based surveillance reduced the removal of healthy, uninfected wildlife and successfully identified three infected mink. Camera trapping showed that there were multiple species present around the farm that were not identified by physical trapping. Both physical trapping and camera trapping have a number of strengths and limitations ((37)). Physical trapping allowed for the collection of biological samples, as well as for the evaluation of the physical condition of the animals; however, physical trapping was labour-intensive and necessitated the euthanasia of trapped animals. Camera trapping was easier to implement and allowed for the collection of a greater quantity of data; however, camera trapping did not allow for the collection of biological samples or for determination of whether the same animal was captured multiple times.

From this specific implementation of wildlife surveillance, a number of considerations have been identified that should inform future surveillance strategies. Factors that should be considered include the species of interest, the season and its impact on the species’ behaviour and lifecycle, the landscape of interest, the practicality of placing and monitoring physical traps or cameras, and the need to collect biological samples to answer the research questions.

## Conclusion

When implementing future surveillance, it is recommended to begin with camera trapping to assess the species present and the frequency of observations. These initial observations can be followed by targeted physical trapping as needed to collect biological samples from specific species of interest. Use of or consultation with experienced wildlife trappers with knowledge of the local area is a critical component and was a significant factor in the success of this project.

## Annex

**Table A1:** Microsatellite loci and polymerase chain reaction conditions used to genotype American mink (*Neovison vison*)

Locus	Final concentration (μM)	Source
Multiplex 1		
Mvi1006 FAM	0.6	((34))
Mvi1016 FAM	0.05	((34))
Mvi075 HEX	0.15	((35))
Mvi1272 HEX	0.25	((36))
Mvi072 HEX	0.1	((35))
Mvi114 NED	0.4	((37))
Mvi002 NED	0.03	((35))
Multiplex 2		
Mvi1321 FAM	0.05	((36))
Mvi1354 FAM	0.5	((36))
Mvi099 FAM	0.2	((35))
Mvi111 HEX	0.15	((37))
Mvi1342 HEX	0.15	((36))
Mvi1302 HEX	0.6	((36))
Mvi2243 NED	0.15	((36))
Mvi4001 NED	0.5	((38))
